# Effects of Low-Intensity Pulsed Ultrasound on New Trabecular Bone during Bone–Tendon Junction Healing in a Rabbit Model: A Synchrotron Radiation Micro-CT Study

**DOI:** 10.1371/journal.pone.0124724

**Published:** 2015-04-15

**Authors:** Hongbin Lu, Cheng Zheng, Zhanwen Wang, Can Chen, Huabin Chen, Jianzhong Hu

**Affiliations:** 1 Department of Sports Medicine, Research Center of Sports Medicine, Xiangya Hospital, Central South University, Changsha, China; 2 Department of Spine Surgery, Research Center of Sports Medicine, Xiangya Hospital, Central South University, Changsha, China; Mayo Clinic, UNITED STATES

## Abstract

This study was designed to evaluate the effects of low-intensity pulsed ultrasound on bone regeneration during the bone–tendon junction healing process and to explore the application of synchrotron radiation micro computed tomography in three dimensional visualization of the bone–tendon junction to evaluate the microarchitecture of new trabecular bone. Twenty four mature New Zealand rabbits underwent partial patellectomy to establish a bone–tendon junction injury model at the patella–patellar tendon complex. Animals were then divided into low-intensity pulsed ultrasound treatment (20 min/day, 7 times/week) and placebo control groups, and were euthanized at week 8 and 16 postoperatively (n = 6 for each group and time point). The patella–patellar tendon specimens were harvested for radiographic, histological and synchrotron radiation micro computed tomography detection. The area of the newly formed bone in the ultrasound group was significantly greater than that of control group at postoperative week 8 and 16. The high resolution three dimensional visualization images of the bone–tendon junction were acquired by synchrotron radiation micro computed tomography. Low-intensity pulsed ultrasound treatment promoted dense and irregular woven bone formation at week 8 with greater bone volume fraction, number and thickness of new trabecular bone but with lower separation. At week 16, ultrasound group specimens contained mature lamellar bone with higher bone volume fraction and thicker trabeculae than that of control group; however, there was no significant difference in separation and number of the new trabecular bone. This study confirms that low-intensity pulsed ultrasound treatment is able to promot bone formation and remodeling of new trabecular bone during the bone–tendon junction healing process in a rabbit model, and the synchrotron radiation micro computed tomography could be applied for three dimensional visualization to quantitatively evaluate the microarchitecture of new bone in bone–tendon junction.

## Introduction

The bone–tendon junction (BTJ) is a complex anatomical structure composed of bone, fibrocartilage and tendon [1, 2]. This unique transitional zone is considered to transmit stress and dissipate stress concentration [3]. Unfortunately, the injury of BTJ is common in trauma and sports exercise, it often occurs in different ligaments, such as rotator cuff, patellar tendon, anterior cruciate ligament, and Achilles tendon [4–6]. Direct surgical reconstruction of bone to tendon is usually indicated for repairing the patella–patellar tendon (PPT) after transverse fractures or comminuted fractures [7, 8]. Since the healing of BTJ includes dissimilar tissues, its repair is slower and more difficult than bone-to-bone or tendon-to-tendon healing [9, 10]. Thus, using a therapeutic approach to enhance the healing of BTJ would be important in basic and clinical research.

As a noninvasive treatment, low-intensity pulsed ultrasound (LIPUS) is considered to be a safe and convenient physical stimulation to administer clinically following musculoskeletal injury [11–15]. It is able to induce osteoblast differentiation, extracellular matrix production, endochondral ossification, and mineralization by mechanical stimulation [16–20]. Our previous studies have shown that LIPUS is able to improve the BTJ healing by enhancing osteogenesis, chondrogenesis, and angiogenesis at the healing interface in a rabbit model [21–23]. We hypothesized that LIPUS would not only be able to promote bone formation but also restore new bone microarchitecture in the BTJ.

Although many detection methods enable observation of the two dimensional (2D) structure of specimens to evaluate bone repair, traditional histology methods with complex section processing steps may destruct the three dimensional (3D) structure of specimens, resulting in limited information to be obtained during and after histological section preparation [24]. In fact, it is impossible to acquire a series of intact and sequential slices using traditional histology methodologies for 3D reconstruction. Therefore, application of a noninvasive and high resolution detection system to evaluate the microarchitecture of new bone is needed. Recently, with the development of imaging technologies and experimental facilities, more internal 3D structural characteristics of specimens could be visualized by noninvasive X-ray computed tomography imaging methods [25, 26]. Compare with the conventional X-ray, the monochromatic X-ray beam produced by synchrotron radiation facility has many advantages, such as high coherent photon flux, brilliant and small angular beam divergence [27, 28]. The synchrotron radiation micro computed tomography (SR-μCT) has received increased attention as a novel imaging measurement with noninvasive and high resolution, able to provide a 3D image and allow quantitative analysis of the microarchitecture of internal structures of tested samples [27, 29–31].

We postulated that LIPUS treatment could promote new trabecular bone formation and remodeling at the BTJ, and the new trabecular bone microarchitecture could be 3D-visualized and quantitatively evaluated using SR-μCT. In the present study, the PPT complexes in the partial patellectomy rabbit model were used for radiographic, histological and SR-μCT detection. The first aim of this study was to evaluate the effects of LIPUS on new trabecular bone during the BTJ healing process in an established partial patellectomy model in rabbits. The second aim was to explore the feasibility of application of SR-μCT in the 3D-visualization of the BTJ, and the quantitative evaluation of the new bone microarchitecture at its healing interface.

## Materials and Methods

All experimental procedures conformed to the Chinese National Health and were approved by the Ethics Committee of the Center for Scientific Research with Animal Models of Central South University (Permit Number: 2012-06-04). All rabbits were maintained under specific pathogen free conditions and fed standard rodent chow ad libitum. The animals were monitored daily for feeding and LIPUS treament throughout the experimental period. All surgery was performed under sodium pentobarbital anesthesia. A pain relief drug (Tramadol, Grunenthal GmbH, Aachen, Germany) and antibiotic (penicillin sodium, North China pharmaceutical Co, Shijiazhuang, China) were administered daily within the first 3 days after surgery, and all efforts were made to minimize suffering. There was no unintended death of animals during this study.

### Animal model and surgery

Twenty four skeletal mature female New Zealand rabbits (3.5 ± 0.2 kg in weight) were provided from the Center for Scientific Research with Animal Models of Central South University. A partial patellectomy according to a previous established experimental protocol was performed on the right hind legs to establish the BTJ injury model [1]. Briefly, the rabbit was anesthetized intravenously with sodium pentobarbital (0.8 ml/kg, Sigma, St Louis, MO), the right hind leg was shaved and the PPT complex was exposed through an anterolateral skin incision. A transverse osteotomy was performed between the proximal 2/3 and the distal 1/3 of the patella. The distal 1/3 of the patella was removed (no remaining fibrocartilage) and two tunnels were drilled longitudinally through the proximal 2/3 patella with a diameter of 0.8mm. The patellar tendon was sutured to the proximal 2/3 patella through two drilled holes with a 3–0 non-absorbable suture (Mersilk, ETHICON Ltd, Edinburgh, UK). A figure-of-eight tension band wire (0.8 mm diameter) was used around the superior pole of the patella and the tibial tuberosity was achieved to preserve surgical reconstruction from overstretching. The knee was immobilized for 4 weeks with a long leg cast with an open window for LIPUS treatment at the resting position. Animals were divided into the LIPUS group (treated with LIPUS, n = 12) and control group (treated with placebo, but no ultrasound, n = 12). Six PPT complexes were harvested from each group at week 8 and 16 postoperatively.

### LIPUS Treatments

Before treatment, animals were anaesthetized with 3% sodium pentobarbital injected by ear vein. An ultrasound signal with a 1.5 MHz frequency, 1:4 duty cycle and 30mW/cm^2^ spatial and temporal average incident intensity was used (Exogen; Smith and Nephew, San Francisco, CA, USA). A daily 20 min/session of LIPUS treatment was given to the surgical animals. The ultrasound signal was delivered by a 2.5 cm diameter ultrasound transducer which was placed on the skin above the surgery site with coupling gel. Treatment was initiated at day 7 and up to week 8 or week16 postoperatively. The control group received placebo treatment only (without ultrasound signal).

### Sample preparation

Animals were sacrificed postoperatively at week 8 or 16 with an overdose of sodium pentobarbital. The PPT complexes were collected using an oscillating hand saw from the operated knee joint of the rabbits. The size of new bone at the BTJ healing interface was evaluated radiographically. After X-ray examination, The PPT complexes were fixed with 4% formaldehyde neutral buffered solution at 4°C for 24 hours, followed by a dual evaporate water rinse for 2 hours. Then, each specimen was sectioned into two halves along the sagittal plane, one half was used for histological detection; the other half was used for SR-μCT scanning.

### Radiographic measurement

Anteroposterior (AP) high-resolution radiographs of the PPT complexes were captured using a Faxitron MX-20 X-ray unit (Faxitron X-ray Corp., Lincolnshire, Illinois, USA) with exposure time at 3 s and tube voltage at 32 kVp. Radiographs were analyzed using an image analysis system (Image-Pro Plus 6.0; Media Cybernetics Inc., Maryland, USA), the size of newly formed bone part from the proximal remaining patella was measured using the previous measurement protocol [1, 22].

### Histology

One half of each specimen was decalcified using 10%EDTA for 3 weeks, followed by a dual evaporate water rinse for 2 hours. Then, specimens were dehydrated using a series of graded ethanols (rinsing in 70, 80, 95 and twice in 100% for 4 hours each) before embedding in paraffin. Specimens were then sectioned (7 μm thick) from the mid-sagittal plane using a microtome (RM2165; Leica Microsystems, Nussloch, Germany), deparaffinized using xylene and stained with hematoxylin-eosin (H&E) for descriptive analysis of the new bone. H&E staining was observed by light microscopy (Olympus CX31; Olympus Inc., Tokyo, Japan).

### SR-μCT imaging and processing

The other half of each specimen was dehydrated using a series of graded ethanols (rinsing in 70, 80, 95 and twice in 100% for 4 hours each) followed by SR-μCT scanning. Scanning experiments were performed at the X-ray imaging and biomedical application beamline (BL13W1) of Shanghai Synchrotron Radiation Facility (SSRF) in China. Each sample was placed at the centre of the rotary stage to allow 180° rotation during scanning. The beam energy was set at 18.0 keV, exposure time was 0.5 s, and the sample-to-detector distance was 5.0 cm. A total of 720 initial projection images were captured by the CCD detector with a pixel size of 3.25 μm. Both dark-field and flat-field images were obtained to reduce the ring artifact side effect during reconstruction. Phase retrieval of the obtained projected images was carried out and these were transformed into 8-bit slices by PITRE software written by BL13W1 [32]. Subsequently, transverse slices were rendered into 3D images using the VG Studio Max software (Ver. 2.1 Volume Graphics GmbH, Germany). The gray-scale images were segmented using a fixed threshold to extract the bone phase followed by a median filter to remove noise. Morphological parameters of the newly formed trabecular bone, such as BV/TV (bone volume fraction), Tb.N (trabecular number), Tb.Th (trabecular thickness), and Tb.Sp (trabecular separation) were calculated.

### Statistical analysis

All quantitative data were given as mean ± standard deviation, and the probability distribution displayed the normal distribution. The results were analyzed statistically using an independent-samples *t* test. Statistical analyses were performed with SPSS 13.0 software, and the significance level was set at *p*<0.05.

## Results

### Measurement on Radiographs

**Fig 1:**
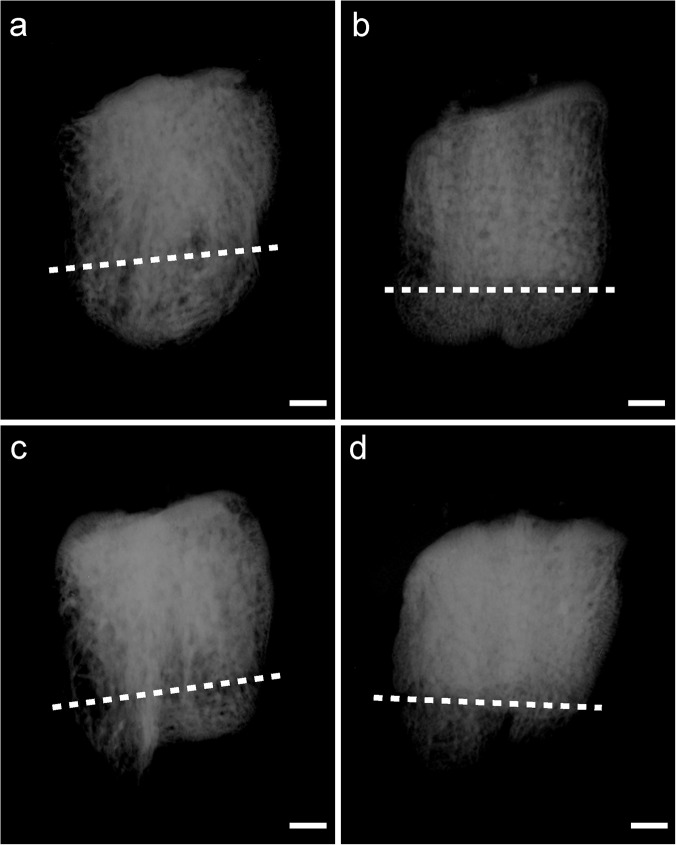
The anteroposterior radiographs of the patella. The area of the new bone increased from week 8 (b, d) to week 16 (a, c) in both groups. Compraed with control (c, d) group, the LIPUS group (a, b) showed more amount of newly formed bone at each time point. The dotted line shows the osteotomy surface. Scanned bar = 1000 μm.

The bone enlarged from the proximal patella at postoperative week 8 and 16. The area of new bone was measured from the AP radiographs (Fig 1). The area of the new bone increased from postoperative week 8 to 16 in both groups. Compared with the control group, there was significantly more newly formed bone in the LIPUS group at postoperative week 8 and week 16 (Table 1).

**Table 1:** The Area of new bone formation (mm^2^).

	Week 8	Week 16
**LIPUS group**	6.71±0.94^a^	9.27±1.27^a^ ^b^
**Control group**	4.63±0.68	6.60±0.99^b^

^a^ *p*<0.01 between LIPUS group and Control group at the same healing time point

^b^ *p*<0.01 between postoperative week 8 and 16 at the same group.

### Descriptive histology

**Fig 2:**
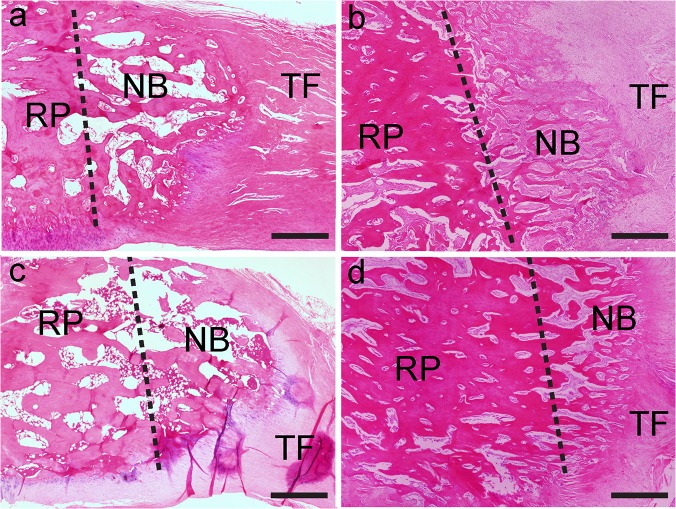
Sagittal H&E-stained sections of the patella–patellar tendon at postoperative week 8 (b, d) and 16 (a, c). New bone formation was found at the osteotomy site in both groups (LIPUS group: a, b; control group: c, d). The dotted line shows the osteotomy surface (NB, newly formed bone; RP, remaining patella; TF, tendon fiber). The LIPUS group showed more advanced remodeling with developed lamellar bone and marrow cavity, which was more distinct and mature. Scanned bar = 1000 μm.

H&E staining showed that the new bone and soft tissue were structurally connected to the remaining patella and tendon at postoperative week 8 and 16 in both groups. Newly formed trabecular bone, with a clear boundary around the osteotomy site, was found at postoperative week 8 in both groups and was less mature compared with those at week 16. As healing time increased from postoperative week 8 to 16, the new trabecular bone pattern changed from woven bone to lamellar bone in the LIPUS group specimens, showing more advanced remodeling with developed lamellar bone and marrow cavity compared with the control group specimens (Fig 2).

### 3D visualization images by SR-μCT

**Fig 3:**
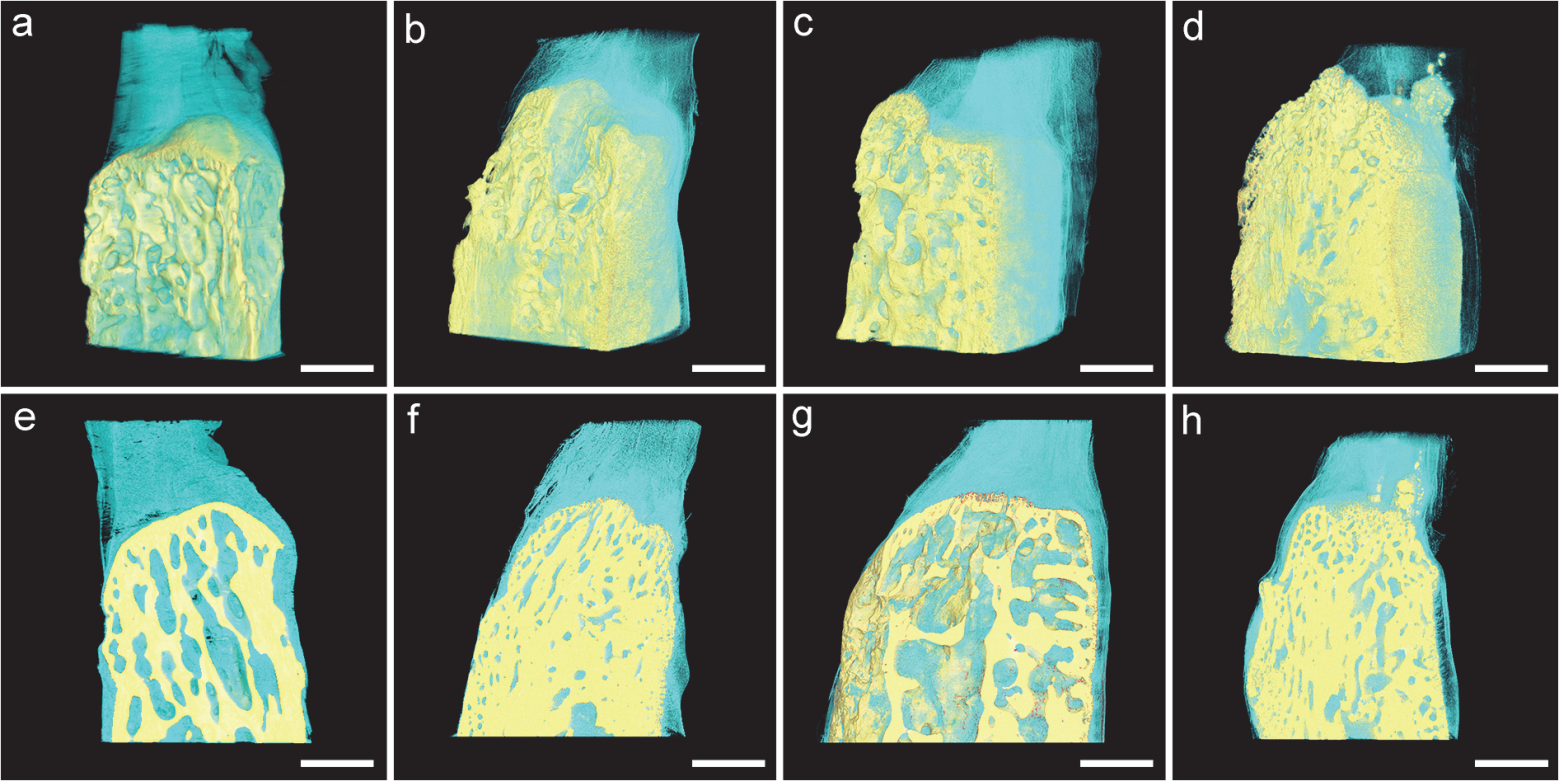
3D visualization images (a–d) and simulation sagittal sections (e–h) of bone–tendon junction of rendered SR-μCT data with high resolution. Primary woven bone was predominant in the LIPUS (b, f) and control (d, h) groups at postoperative week 8. At postoperative week 16, lamellar bone with marrow cavity formation was predominant in the LIPUS (a, e) and control (c, g) groups. Bone (yellow) and tendon (green) tissues could be clearly distinguished, and the marrow tissue was also seen in green. Scanned bar = 1000 μm.

Because of the high resolution and signal-to-noise ratio of SR-μCT, 3D visualization images of the BTJ were acquired, which clearly distinguished the mineralization bone phase, tendon and interface between them (Fig 3), the sample resolution was about 6.5 μm. The maximum size of field of view was 6.656 mm under the pixel size of 3.25 μm of CCD in BL13W1 of SSRF. Imaging of the whole patella length was not possible because of the limitation of field of view. However new bone was observed in all specimens within each field of view. As healing time increased, new bone gradually formed on the remaining patella and reconstruction was observed in both groups. Based on the simulation sagittal sections, the visualization images of trabecular bone in both groups were well matched following H&E staining, and and showed that the trabecular bone at postoperative week 16 in the LIPUS group had more lamellar bone with an enlarged marrow cavity arranged in regular orientation.

**Fig 4:**
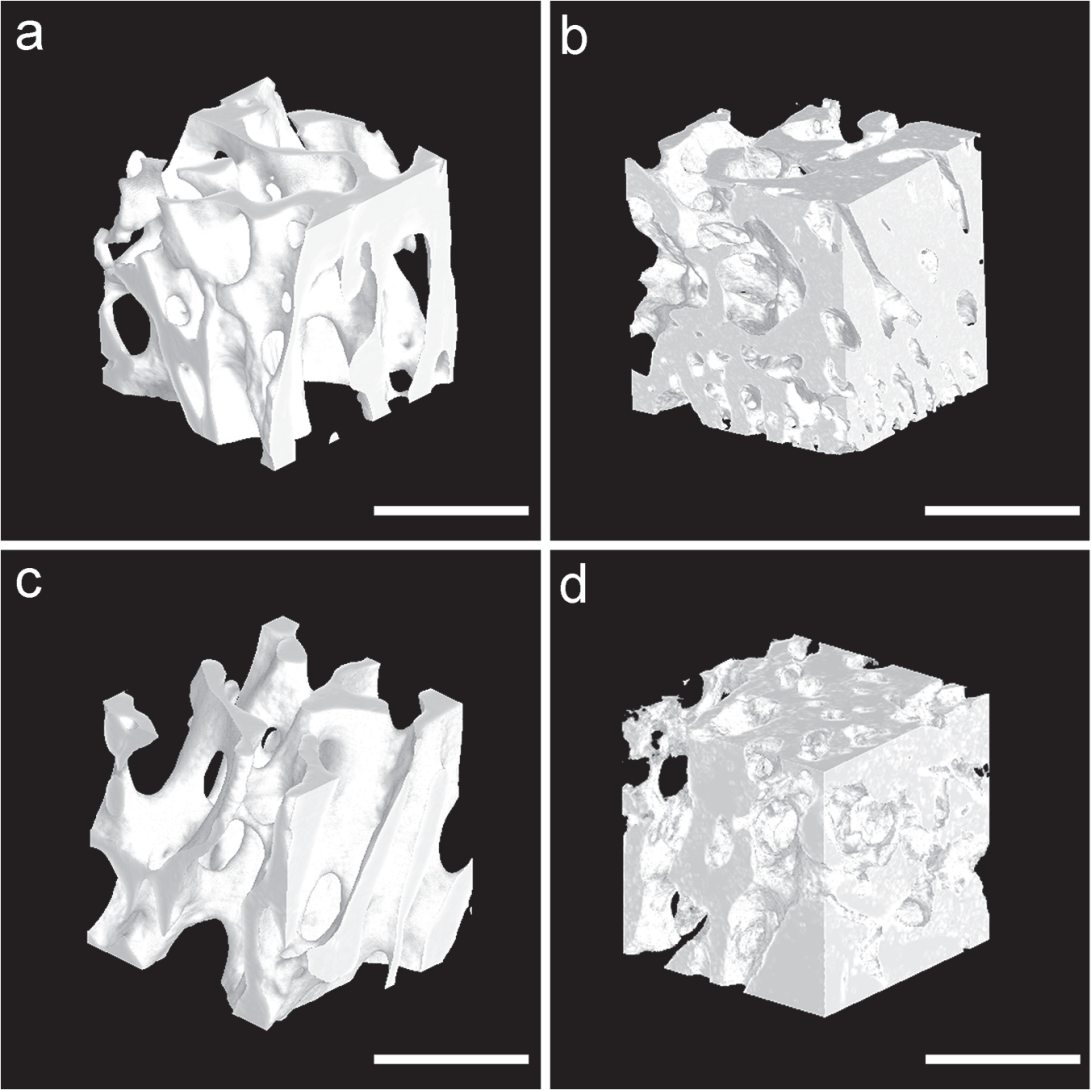
Segmented 3D tomographic reconstruction images of newly formed trabecular bone in the region of interest. The new trabecular bone was dense with crisscross arrangement in the LIPUS (b) and control (d) groups at postoperative week 8. At postoperative week 16, the new trabecular bone was sparse with marrow cavity formation in the LIPUS (a) and control (c) groups. Scanned bar = 1000 μm.

**Fig 5:**
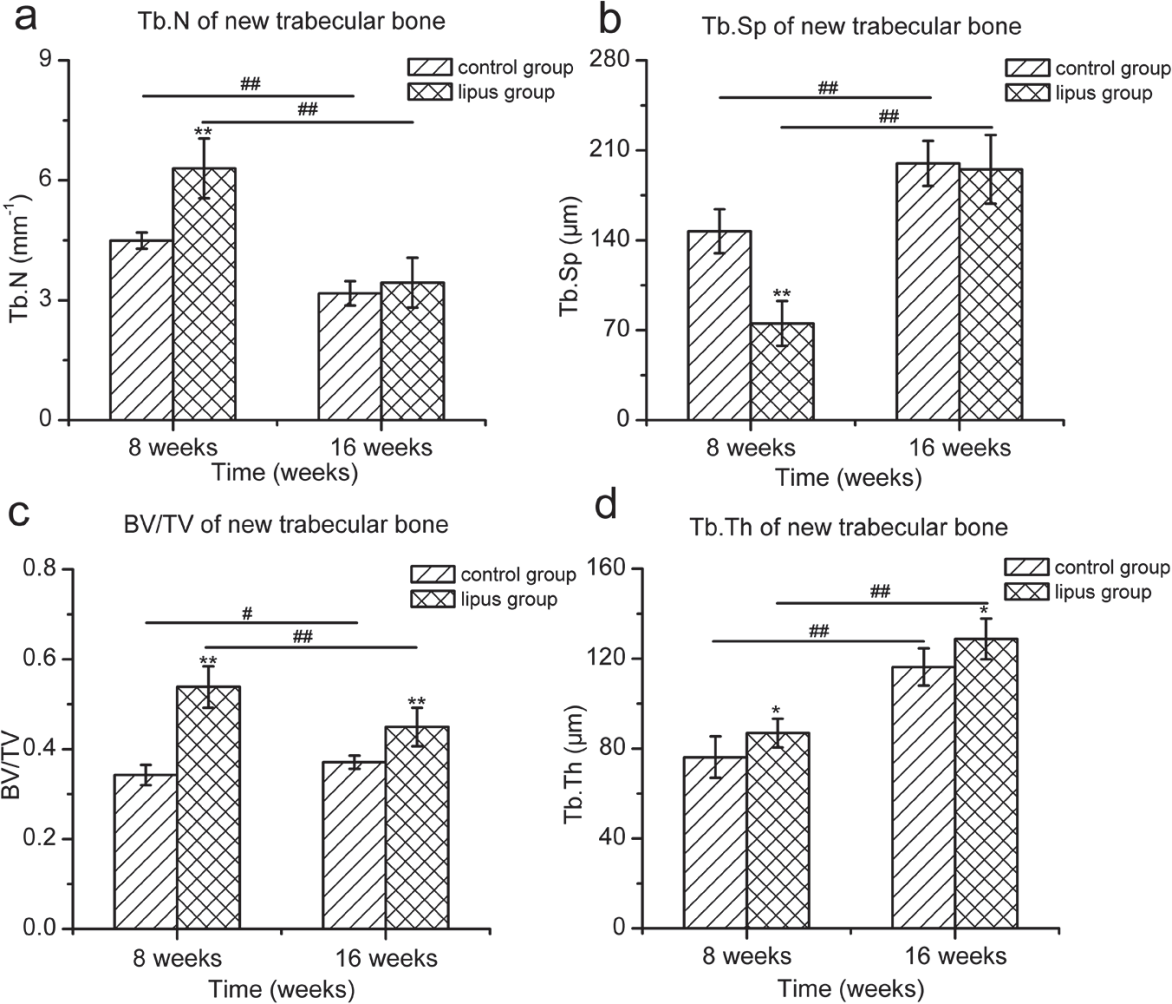
Histogram of morphological parameters of newly formed trabecular bone. The data show that bone formation and remodeling in the LIPUS group were more advanced than in the control group (a: Tb.N; b: Tb.Sp; c: BV/TV; d: Tb.Th). (*p<0.05; **p<0.01, the difference between the LIPUS group and the control group at different time points; #*p*<0.05; ##*p*<0.01, the difference between postoperative week 8 and 16 for the LIPUS and control group; n = 6 for each group/time point).

After selecting the region of interest (ROI) of newly formed trabecular bone, bone tissue was segmented and vivid 3D images were acquired (Fig 4). In order to reduce the effect of the region, the ROI was located in the central of newly formed trabecular bone. Morphological parameters of each ROI were analyzed (Fig 5). At postoperative week 8, the BV/TV, Tb.Th and Tb.N of new trabecular bone in the LIPUS group were significantly higher than those of the control group (BV/TV: 0.54 ± 0.05 vs 0.34 ± 0.02, *p*<0.01, Tb.Th: 86.96 ± 6.38 vs 76.16 ± 9.22 μm, *p*<0.05, Tb.N: 6.30 ± 0.75 vs 4.49 ± 0.20 1/mm, *p*<0.01), the Tb.Sp of new trabecular bone in LIPUS group was significantly lower than that of the control group (75.27 ± 17.43 vs 147.02 ± 17.14 μm, *p*<0.01). At postoperative week 16, the BV/TV and Tb.Th of new trabecular bone in the LIPUS group were significantly higher than those of the control group (BV/TV: 0.45 ± 0.04 vs 0.37 ± 0.02, *p*<0.01, Tb.Th: 128.78 ± 39.10 vs 117.98 ± 9.02 μm, *p*<0.05). There was no significant difference in the Tb.N and Tb.Sp between the two groups (Tb.N: 3.44 ± 0.63 vs 3.17 ± 0.31 1/mm, *p*>0.05, Tb.Sp: 195.13 ± 26.75 vs 199.83 ± 17.54 μm, *p*>0.05). With the healing time increased, the BV/TV of new trabecular bone increased from postoperative week 8 to 16 in the control group (*p*<0.05), and decreased in the LIPUS group (*p*<0.01). The Tb.Th and Tb.Sp increased from postoperative week 8 to 16 in both groups (*p*<0.01), and the Tb.N decreased from postoperative week 8 to 16 in both groups (*p*<0.01).

## Discussion

The present study was designed to investigate the effects of LIPUS on new trabecular bone formation during the BTJ healing process in an established partial patellectomy model in rabbits, and to explore the feasibility of application of SR-μCT for 3D visualization of the BTJ and quantitative evaluation of the microarchitecture of new bone at the healing interface. The histologic and SR-μCT results demonstrated that LIPUS accelerated new bone formation and remodeling during the BTJ healing process, and SR-μCT could be applied for 3D visualization and quantitative evaluation of the microarchitecture of new bone.

The rabbit PPT complex in a BTJ injury model, established by partial patellectomy, was a good model to study the BTJ healing process [1]. In the present study, more new bones were formed distally from the osteotomy site of the remaining patella in the LIPUS treatment group as compared with the control group. The osteogenic effects of LIPUS have been confirmed by both *in vitro* and *in vivo* studies [17, 33]. Previous research has found that new bone formation of the remaining patella increased the patellofemoral contact area after partial patellectomy, which decreased the contact pressure [34]. New bone formation is the foundation of functional recovery after BTJ injury [35]. Our previous study found that the size of new bone at the junction during BTJ healing correlated with the functional properties (Failure load, Ultimate strength and Energy at failure) [36], and LIPUS was able to enhance BTJ repair in a partial patellectomy model in rabbits, especially accelerated bone formation [22, 23]. Thus, we believed that LIPUS treatment could promote new bone formation and play a positive role during the BTJ healing process.

Better remodeling of the new trabecular bone occurred in the LIPUS treatment group when compared with the control group. Based on the H&E staining and SR-μCT data, the newly formed trabecular bone pattern changed from dense and irregular woven bone at the early stage to mature lamellar bone and formation of a marrow cavity at the late stage of the healing process. In the present study, the new trabecular bone was dense with a crisscross arrangement in both groups at postoperative week 8. Furthermore, the bone volume fraction and number and thickness of new trabecular bone in the LIPUS group were greater with lower separation than those of the control group. At postoperative week 16, the new trabecular bone was thicker and had sparse marrow cavity formation, and when compared with the control group, the bone volume fraction and thickness of new trabecular bone were greater in the LIPUS group.

Bone healing is usually composed of four distinct but overlapping stages: 1) the early inflammatory response; 2) soft callus formation; 3) hard callus formation and 4) late bone remodeling [37]. The remodeling process of new bone is crucial to restore the function of the damaged structure [38], and it is accomplished during the remodeling stage in which the irregular and disorderly woven bone callus converts into lamellar bone with orderly bone resorption, eventually restoring the original trabecular bone configuration, structure and mechanical properties [37, 39, 40]. In the present study, quantitative analysis of the morphological parameters showed that the osteogenic effect was more active in week 8 during the healing process, and the remodeling of new trabecular bone after week 8 was more advanced in the LIPUS group. Although the amount of new bone was augmented with healing time, the bone volume fraction of the new trabecular bone decreased with greater internal microarchitecture variation. For example, the new trabecular bone was thicker with regular arrangement, and the separation was enlarged with marrow cavity formation. These results suggested that LIPUS was also able to accelerate bone remodeling, which occurred more slowly in the control group.

The three-dimensionality of biological tissue is important, and new bone is augmented with 3D direction in biological tissue during the repair process. To observe the internal microarchitecture of new trabecular bone at the BTJ healing interface, we successfully acquired the 3D visualization images, including the bone, tendon and interface between them with a pixel size 3.25 μm using SR-μCT without complex sample preparation procedures. Based on the high resolution 3D images of the BTJ, the simulation sections were highly related to the traditional histology slices without structure destruction, and were not restricted by angle and position.

In addition, we not only obtained high-definition 3D images of the internal microarchitecture of new trabecular bone in the BTJ samples by SR-μCT, but also performed quantitative analysis to evaluate the effects of LIPUS on new trabecular bone during the BTJ healing process using morphological parameters. With the development of imaging technology, the functions of imaging software have been constantly improving. Therefore the high resolution and 3D images of different tissues in biological samples obtained by SR-μCT enable more detailed information to be observed from any angle and orientation using imaging software during the imaging process. In recent years, 3D visualization the images of microarchitecture of human and animal bone specimens at different sites were shown using SR-μCT, noninvasively and at high resolution, such as the femur, tibia and vertebrae [26, 30, 41–47]. Microarchitecture characterization of bone within different bone diseases, including osteoporosis[48], osteoarthritis[43] and bone metastases[42], have also been carried using SR-μCT, which was also used to predict bone fragility or microcracks [29]. Although the microarchitecture index could be obtained by SR-μCT, there was a lack of research for using this method as an evaluation tool to study the effects of treatment or intervention. To our knowledge, this is the first study to performe 3D visualization of new trabecular bone microarchitecture in the BTJ and evaluation of the effects of LIPUS on new trabecular bone during BTJ healing in a rabbit model using SR-μCT.

In this study, some limitations exist. SR-μCT detection is not easy to perform because the facility is too large and expensive for scientific research and clinical application. Furthermore, the number of facilities and the size of field of view are also limited. However, with the development of imaging technologies, we believe that these limitations will be solved prior to clinical application.

## Conclusions

LIPUS treatment was able to promote BTJ healing by accelerating bone formation and remodeling of new trabecular bone during the healing process in a rabbit model. SR-μCT was successfully applied for 3D visualization and quantitative evaluation of the microarchitecture of new bone in the BTJ.

## Supporting Information

S1 ARRIVE Checklist ARRIVE Guidelines Checklist. (PDF)
