# Correction: Effects of Low-Intensity Pulsed Ultrasound on New Trabecular Bone during Bone-Tendon Junction Healing in a Rabbit Model: A Synchrotron Radiation Micro-CT Study

**DOI:** 10.1371/journal.pone.0214974

**Published:** 2019-04-01

**Authors:** Hongbin Lu, Cheng Zheng, Zhanwen Wang, Can Chen, Huabin Chen, Jianzhong Hu

Fig 4 is incorrect. The authors have provided a corrected version here.

**Fig 4:**
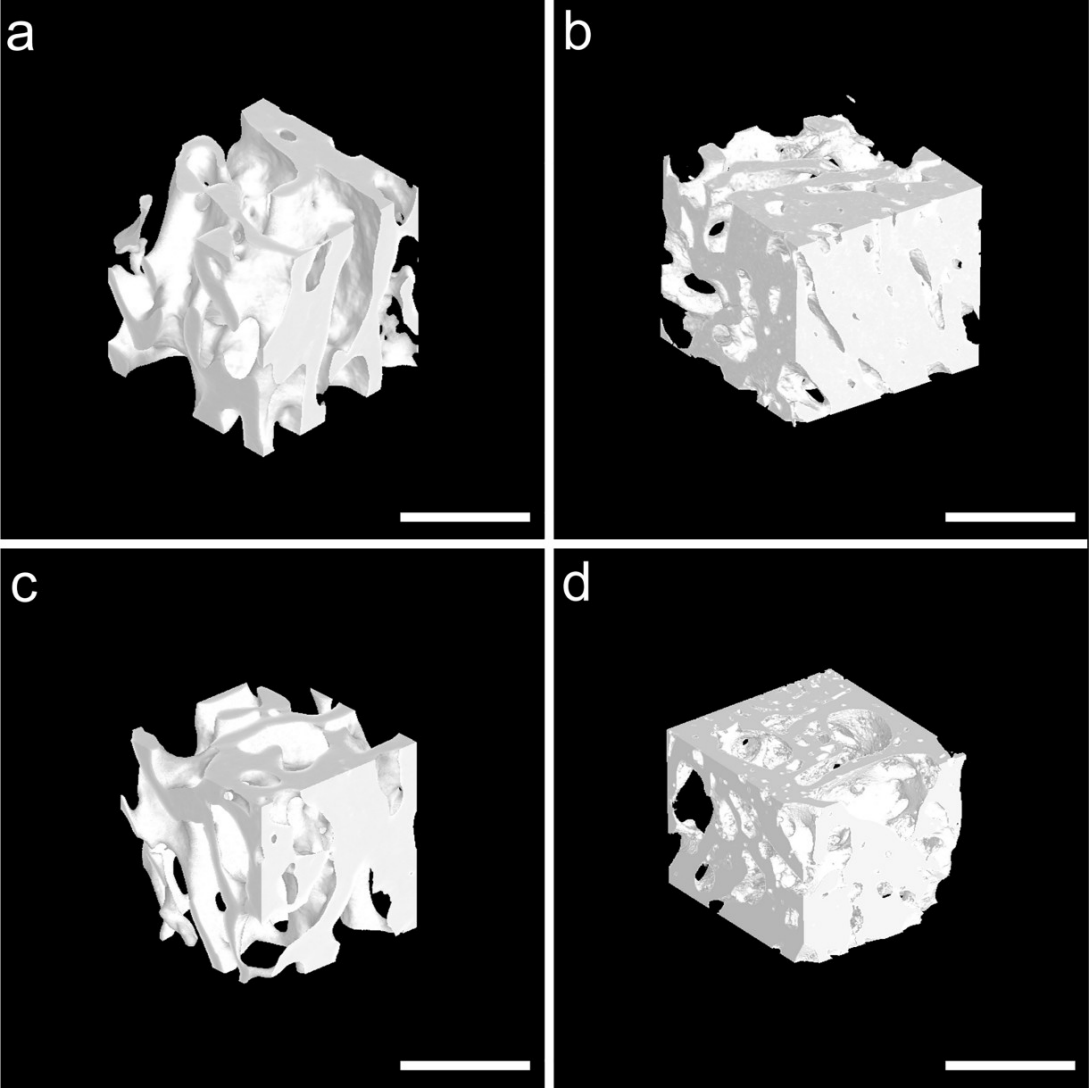
Segmented 3D tomographic reconstruction images of newly formed trabecular bone in the region of interest. The new trabecular bone was dense with crisscross arrangement in the LIPUS (b) and control (d) groups at postoperative week 8. At postoperative week 16, the new trabecular bone was sparse with marrow cavity formation in the LIPUS (a) and control (c) groups. Scanned bar = 1000 μm.
